# Supplementary material for: Health services costs for ovarian cancer in Australia: Estimates from the 45 and Up Study
Source: PLoS One. 2023 Apr 18;18(4):e0282851. doi: 10.1371/journal.pone.0282851 (PMC10112806; doi:10.1371/journal.pone.0282851)
Supplement: Attachment — Submitted filename: Response to Reviewers.docx [file pone.0282851.s001.docx]

**Reviewer #1**

*Comment: Your paper entitled “Health services costs for ovarian cancer in Australia: estimates from the 45 and Up Study” is very relevant since health care costs of cancer are increasing and there is a large debate about the sustainability of health care systems in all developed countries. The evidence you provided might contribute to address the health care policies in order to reduce ovarian cancer burden. While this is a valuable contribution to the field, I have some suggestions to improve the clarity of your paper and the impact of results and interpretation.*

*Major points*

*1) According to the methods-data source section, your results are based on the Sax Institute’s 45 and Up study which includes data from 18% of individuals invited to participate in the study, they represent 11% of the NSW population aged 45+. Please add a comment in the Discussion section about the issue of the response rate of the 45 and Up Study, which influences the representativeness of the study cohort and by the consequence the transferability of the cancer cost estimations to the Australian general population;*

*Response*: Thank you for the thoughtful and constructive feedback. We have added the below underlined text to the section about representativeness in the Discussion:

“However, there were some differences between the cases in this cohort and all ovarian cancer cases in Australia. … While the ~~The~~ 45 and Up Study cohort ~~is~~ achieved an 18% response rate during recruitment, participants are overall healthier than the general NSW population [19]~~, which may also have implications for overall cost estimates~~. Thus, 45 and Up Study participants with ovarian cancer may also not be representative of all people with ovarian cancer in Australia. We have re-weighted results to match the state-wide stage distribution to improve generalisability, however caution should be used in extrapolating results to the whole-of-population level in Australia.”

*Comment: 2) A major limitation of this study is the update of the cost information since the study sample includes women diagnosed with ovarian cancer up to 2013. This implies that new treatments, particularly pharmaceutical ones, are not taken into account. However, it is not clear if all high cost drugs and target therapies are subsidized by the PBS (Pharmaceutical Benefits Scheme) or not, please add a sentence clarifying this point in the methods-data source section;*

*Response*: The Methods section has been revised to clarify this point as follows:

“…costs were based on individuals’ inpatient hospital and emergency department records (APDC and EDDC, respectively), subsidised prescription medicines captured in the PBS and subsidised medical services captured in the MBS. Only drugs and therapies that were subsidised through the PBS or MBS during the period of Jan 2006 - Jun 2016 were included in the total healthcare system costs. Data was not collected for other costs associated with ovarian cancer during the study period including off-label medications.”

We have also addressed this aspect in the Discussion section:

“This study captured costs associated with ovarian cancer treatment for women diagnosed up to 2013. However these reported costs will likely have changed since that time, through changes in clinical recommendations, licencing of new medications and technologies, and their utilisation and uptake through the health system [27, 28]. For example, changes occurring during the health care utilisation period of this study include PBS listing of bevacizumab in 2014, although there was very little take-up in this cohort.”

*Minor points*

*Comment: 3) In the methods section you reference to a previous paper published in 2018 by Goldsbury et al for more details about the methods to extrapolate results from the 45 and Up study to the whole Australia in 2013, however a very short description of the methodology used would help the reader to interpret the results;*

*Response*: The Methods section has been revised with the description shown below.

“We estimated the total healthcare costs for ovarian cancer incurred in Australia in 2013 for incident and prevalent cancers diagnosed in 2009-2013, using annual excess costs per case, and population-wide incidence~~, mortality,~~ and survival rates for 2009-2013 as described previously [16]. In brief, we first considered the national number of ovarian cancer cases diagnosed in 2013 and applied the annual per-case costs for the year after diagnosis. Then we considered the national number of ovarian cancer cases diagnosed in 2012, used the 1-year survival to calculate the number of these alive in 2013, and applied the mean costs for the second year after diagnosis. We repeated the process up to new cases diagnosed in 2009, with 4-year survival and mean costs for the fifth year after diagnosis. The totals were combined to estimate the total healthcare costs for 2013.”

*Comment: 4) Stage at diagnosis is demonstrated to be a cost driver, especially in the initial phase of the disease. However results by stage in the terminal phase of care are incomplete due to the low number of deaths in localized and regional cases (see Figure 2). A comment on the expected trend by stage in the terminal phase of care compared to the continuing and initial phases should be included in the discussion;*

*Response*: We have added the below text to the cohort description in the Discussion section.

“Furthermore, there were relatively few patients with localised/regional stage ovarian cancer with terminal phase information. Our prior work in colorectal and lung cancer found no clear trend in terminal phase costs by cancer stage at diagnosis [44,45], and this could also be the case for ovarian cancer. A limitation of our dataset was that it precluded us performing detailed analysis on this aspect of ovarian cancer costs.”

*Comment: 5) It is not clear if and how you used the information about the cause of death with respect to the terminal phase of care definition: are those cases dead for causes other than cancer treated as censored cases of the continuing phase of care? are they considered eligible for the terminal phase just because they died? Please clarify;*

*Response*: We have clarified this in the Methods section as shown below.

“For patients who died of any cause before July 2016, the final year up to and including the death date was designated the terminal phase.”

*Comment: 6) Figures 2 and 3 are blurred and results are not clear, there is no contrast between different categories using black and white printing, I suggest to add textures and/or labels in the box-plot and histograms in order to make them more readable;*

*Response*: These Figures have been adjusted in the revised version.

*Comment: 7) In the results section, page 12, lines 245-247, you make reference to Table 2 and indicate: “The mean cost from one year pre-diagnosis until two years after diagnosis was approximately $58,000 per patient, including those who died during the period”. However, from Table 2, the mean cost is about $61,000, summing up the mean costs by phase of care of the overall category: how do you explain this inconsistency?*

*Response*: These are two separate calculations with slightly differing denominators. The values shown in Table 2 reflect the average costs for those alive in the specific period pre- or post-diagnosis, as per the table footnote (“Includes women alive at the start of each time period”). By contrast, the figure of $58,000 per patient is an average cost up to 2 years post-diagnosis for all those diagnosed with ovarian cancer regardless of survival time, so it will include some people who died in the first year after diagnosis, and who would not be included in the “>1-2 years post-diagnosis” calculation in Table 2. We have clarified this in the Results section as follows:

“The mean cost from one year pre-diagnosis until two years after diagnosis was approximately $58,000 per patient, including those who died in the first year after diagnosis ~~during the period~~.”

*Comment: 8) In the results section, page 15, lines 283-287, there is the total number of prevalent cases with ovarian cancer in Australia in 2013 and the corresponding total direct health care costs estimated from data of the 45 and Up study, it would be interesting to report the distribution of these cases and costs by phase of care.*

*Response*: We thank the reviewer for this suggestion. We have included the distribution of cases and costs by phase of care in the Results section as follows:

“Based on the number of all ovarian cancers diagnosed in Australia and the national survival rates, we estimated that 4,700 women diagnosed in Australia in 2009-2013 were alive in 2013, with 23% in the initial phase, 61% in the continuing phase, and 17% in the terminal phase. Using the annual excess costs estimated in this study, the overall direct healthcare costs of ovarian cancer in these women totalled 2013AUD$99 million in 2013 alone (equivalent to 2021AUD$132 million based on the health index of the Australian consumer price index), with 40% of costs for people in the initial phase, 25% for those in the continuing phase, and 35% for those in the terminal phase.”

This has also been noted in the Methods section as follows:

“We estimated the total healthcare costs for ovarian cancer incurred in Australia in 2013 … . The totals were combined to estimate the total healthcare costs for 2013. The same process was used to assign people to their phase of care during 2013, and the corresponding phase costs were applied.”

**Reviewer #2**

*Comment: I am somewhat* *doubtful of the utility of this study as it reports on costs prior to the introduction of new treatments which are now considered standard care. The authors make the argument that their cost data can be used for health economic evaluations but I disagree as ‘usual care’ is not captured in their data. I am struggling to see how this paper adds something new and useful to the literature in this field.*

*Response*: The primary aim of the research was to provide a benchmark for the costs of ovarian cancer care in Australia, with comparisons across key patient subgroups. While this work uses data for cancers diagnosed to 2013 and treated to 2016, these are the most recent data that were available, and these estimates fill an important gap given the scarcity of population-based ovarian cancer cost estimates for Australia. Moreover, these benchmark estimates can be used to evaluate the health economic impacts of the new treatments (as noted in the Discussion).

Thus, we believe these results are still of interest to a range of stakeholders. We have previously found that benchmarks of costs prior to the introduction of new treatments or cancer control interventions can be important and impactful, especially from a large-scale, population-based dataset with comprehensive linked data and a rigorous methodology. As an example, our previous paper on lung cancer costs estimated based on the same study period [reference 44] was used by the Australian government to support the economic case for lung cancer screening, despite it not capturing recent changes in the standard of care (since 2016, at least two new lung cancer therapies have been added to the PBS-subsidised list each year, with more on the horizon). And as noted above, costs for new therapies can be added to these benchmarks in the future.
