# Supplementary material for: Health services costs for ovarian cancer in Australia: Estimates from the 45 and Up Study
Source: PLoS One. 2023 Apr 18;18(4):e0282851. doi: 10.1371/journal.pone.0282851 (PMC10112806; doi:10.1371/journal.pone.0282851)
Supplement: Attachment — Submitted filename: Response to Reviewers.docx [file pone.0282851.s002.docx]

**Responses to review of revised manuscript**

**Reviewer #2**

*Comment: Please state the perspective of the study up-front.*

*Response*: The abstract has been adjusted as follows:

“This study estimated the direct health system costs (government perspective) for women diagnosed with ovarian cancer in Australia.”

We have also adjusted the Introduction section:

“Therefore, the aim of this study was to estimate the per case excess direct health services costs (government perspective) for women diagnosed with incident ovarian cancer.”

*Comment: Can the authors comment on the impact of only including women aged 45 and older in the analyses? Do younger women incur similar costs or are there different treatments indicated for ovarian cancers diagnosed at earlier ages?*

*Response*: Women aged <45 years at diagnosis account for 11% of all ovarian cancers in Australia and thus from the perspective of age distribution, our data represent the large majority of ovarian cancer cases. As described in the Discussion section, the sample median is similar to the population-wide median, and we have added a note about the potential impact of only including women aged 45 and older.

“The median age at diagnosis in the study sample was 68 years, which is slightly older than a previous national report of 65 years [17]. This is likely due to the age of recruitment into the study (age 45 and above) and therefore exclusion of women who develop ovarian cancer at a younger age, although women aged <45 years account for 11% of incident ovarian cancers in Australia so it would have limited impact overall [44]. Younger cases are more likely to have early stage and/or non high-grade serous cancers and may have fertility-sparing treatment. Overall, those aged <40 years account for only 5% of hospitalisations [17] and thus might have lower treatment costs.”

*Comment: Why was Cancer Registry data only available up to 2013? From experience, more recent data is normally available.*

*Response*: The data were part of a wider data linkage project that included several large data sources, and at the time of analysis these were the most recent data available to us. This has now been noted in the Discussion section. Unfortunately, in the setting in which the work was conducted (NSW, Australia), the availability of cancer registry data has regularly been delayed for several years. Although data after 2013 were not available, this still represents a major advance given the current absence of published data on ovarian cancer costs in Australia.

“This study captured costs associated with ovarian cancer treatment for women diagnosed up to 2013, as these were the most recent data available to us at the time of analysis.”

*Comment: Table 2: it would be useful to include 95%CI or SDs for context. Also, can the authors comment on why mean excess costs in the year preceding diagnosis were higher.*

*Response*: Table 2 has been revised to include the standard deviations. Regarding the excess costs in the year prior to diagnosis, further analysis found they are largely related to diagnostic/testing procedures and consultations in the lead-up to diagnosis. We have noted this in the Results as follows:

“Mean excess costs were largest in the year following diagnosis, at $42,469 per person, with costs generally decreasing each subsequent year. Excess costs in the year prior to diagnosis were commonly for imaging procedures (e.g. CT scans, ultrasounds) and specialist consultations.”

*Comment: Did the authors consider modelling the effect of socioeconomic and clinical variables on the costs?*

*Response*: We performed this type of more detailed analysis for previous studies of colorectal cancer and lung cancer, where we had larger samples to provide more statistical power for regression analyses. Unfortunately we did not have a large enough sample to enable more detailed statistical analyses here. However we still included analyses by phases of care and sources of costs, and have reported costs by stage of disease, to give some indication of cost variation.

*Comment: As many of the new therapeutic agents for cancer treatment are high cost, it would be useful to understand the impact these have had on overall costs.*

*Response*: The Discussion section included two paragraphs on new and evolving therapeutic agents, noting that there was virtually none included in our study period and one estimated cost impact. We have added some information on the potential cost impact of the additional genetic testing.

“Since the study period, recommendations for *~~BRCA1/2~~* genetic testing have evolved to include most women with ovarian cancer [29, 30], mainstreaming of genetic testing, and inclusion of a test to detect germline pathogenic *BRCA1* or *BRCA2* genetic variants in the MBS from 2017, at a cost of ~$1,000 per eligible person [31]. There are also new recommendations for the use of the PARPi, olaparib, based on the results of this test. Olaparib was not listed on the PBS during the study period but is now PBS listed and subsidised for women with newly diagnosed ovarian cancer who have a *BRCA* pathogenic variant [32]. This inclusion will result in additional direct health service cost of approximately $6,890 per eligible person per month [33].”

*Comment: The Discussion states: Our previous cross-cancer study using the same methods and data sources as this work found that average costs of all cancers by phase were $28,719 for the initial treatment phase, $4,474 per year in the continuing care phase and $49,733 for the terminal care phase [16], compared with $40,556 (+41%), $9,514 (+113%) and $49,208 (-1%), respectively, for ovarian cancer presented here. It would be appreciated if the authors can present these results with a measure of dispersion.*

*Response*: We have included the all-cancer standard deviations as follows. The corresponding figures for ovarian cancer were included in Table 2 as described above.

“… average costs of all cancers by phase were $28,719 (SD $32,151) for the initial treatment phase, $4,474 (SD $17,390) per year in the continuing care phase and $49,733 (SD $49,861) for the terminal care phase [16] …”

*Comment: Also in the Discussion: “Interestingly, the annual costs during the continuing care phase for ovarian cancer appeared twice as high as the estimated average of all cancers. This is partly due to the relatively high proportion of women with distant metastatic disease at diagnosis (~60%), who have higher continuing care phase costs ($13,814 compared to $4,884 for localised/regional).” This difference is likely due to the selection of a 12-month cut-off for the initiation phase. Can the authors please explain why this was selected. Is this an arbitrary cut-off? Maybe this is unsuitable for ovarian cancer given the high rate of patients with metastatic spread.*

*Response*: We used 12 months for the initial treatment phase for consistency with our earlier work (refs 16,45,46), which was based on a previous US study (Mariotto et al., *JNCI* 2010), as well as to align with other reporting of cancer treatment patterns in a way that would work best across all cancer types. A cut-off needed to be selected that could be used for all cancer types, and the cut-offs are explained in the Methods so that the results can be interpreted and/or applied appropriately. Given the noted difference in spread of disease, we have also added the following in the Discussion.

“This is partly due to the relatively high proportion of women with distant metastatic disease at diagnosis (~60%), who have higher continuing care phase costs ($13,814 compared to $4,884 for localised/regional). The continuing care phase costs might also be higher for ovarian cancer due to the relatively high rate of recurrence and progression to metastatic disease.”

*Comment: Given the relatively high rates of surgical complications and adverse events for this patient group, could the authors look at how this contributed to costs? Surgical complications may be identifiable through use of Condition Onset Flag in the APDC, or a subsequent admission within 30 days that was related to the index admission.*

*Response*: This is an interesting suggestion but would require a complicated analysis that we feel is unlikely to give meaningful results from our particular dataset. The condition onset flag indicates whether any of the diagnoses at the hospitalisation were “noted as arising during the episode of admitted patient care”. Checking all recorded admissions for people with cancer in our dataset, this was flagged at 5.2% of admissions, with similar levels pre- and post-diagnosis, and it was flagged at 3.5% of admissions for cancer-free controls. However, there are generally multiple diagnoses at each admission. To accurately assign complications to treatment for ovarian cancer, we would need to check all recorded diagnoses and procedures, and correctly identify which ones were attributable to the treatment for ovarian cancer. This detailed analysis is beyond the scope of this study, and highlights a strength of the excess costs method, whereby a broad comparison with matched cancer-free controls gives an indication of what can be attributed to the condition of interest.
